# Exploring poems of intersectionality in the disorientation of interprofessional learning

**DOI:** 10.1007/s10459-025-10428-5

**Published:** 2025-03-31

**Authors:** Jana Müller, Abigail Dreyer, Elize Archer, Ian Couper

**Affiliations:** 1 Division of Rural Health (Ukwanda), Department of Global Health, Faculty of Medicine and Health Sciences, Stellenbosch University, Cape Town, South Africa; 2 Department of Health Professions Education, Faculty of Medicine and Health Sciences, Stellenbosch University, Cape Town, South Africa

**Keywords:** Interprofessional education, Intersectionality, Transformative learning, Poetic inquiry, Health professions education

## Abstract

Exploring students’ interprofessional education experiences highlights the occurrence of hierarchy as a barrier to collaborative practice. Individuals are however influenced by the multiple social identities of themselves and others and not just professional hierarchy. Intersectionality offers a useful lens through which to understand the complex influences of students’ learning experiences. Using poetic inquiry, this paper explores the influence of intersectionality on health professional students’ interprofessional learning experiences on two rural training platforms in South Africa. Sixteen individual interviews with final-year undergraduate students from five different healthcare professions were conducted in 2022. An inductive narrative analysis of the data was undertaken and represented using ‘found poems’. Reflexive analysis of the data presented in poems was conducted with student participants, co-authors, and an independent qualitative researcher. Themes related to the intersection of language and ethnicity, religion and profession, culture and profession as well as professional discipline and being a student were extracted from the data. Participants demonstrated disorienting learning experiences in both the clinical and social context. Using intersectionality as a lens, we have gained insight into the sometimes-disorienting influence of students’ intersecting social identities during interprofessional learning on two rural training platforms. A nuanced understanding of how multiple social identities intersect to influence experiences could help educators mitigate student and educator biases and understand structural power dynamics in training environments. Transformative learning may be a way to introduce intersectionality into both interprofessional education and health professions education in general.

**Supplementary Information:**

The online version contains supplementary material available at 10.1007/s10459-025-10428-5.

## Introduction

Interprofessional education (IPE) is considered to take place when students learn with, from and about each other, to improve collaboration and the quality of care (Canadian Interprofessional Health Collaborative, 2010). Multiple factors influence the efficacy of IPE, such as the coordination of student schedules, availability of facilitators, and existing professional hierarchies (Bogossian et al., 2023; Khalili et al., 2022). Research related to the outcome of IPE has predominantly centred around students’ readiness to engage interprofessionally, their intent to do so, and the presence of interprofessional attributes (Brandt et al., 2014; Kirkpatrick et al., 2023; Reeves et al., 2016). Interprofessional learning extends beyond IPE and includes serendipitous spaces. However, IPE (formalised learning experiences) and interprofessional learning (informal interprofessional experiences)are also influenced by individuals’ gender, ethnicity, and culture (Bridges et al., 2011; Olson et al., 2016; Yamuragiye et al., 2021). These are important aspects to consider when designing interprofessional learning spaces and experiences during training.

All individuals have multiple social identities, such as gender, ethnicity, language, and profession amongst others, that are interrelated and influence experiences. One identity should not foreground another, but be considered in the complex dynamic between person and context (Tsouroufli et al., 2011). The intersection of students’ social identities along with their own and others’ historical biases and assumptions influence their engagement in learning (Crampton & Afzali, 2021; Tsouroufli et al., 2011; Wyatt et al., 2021). For this reason, no two people’s experiences of discrimination or marginalisation are alike, and every learning experience depends on the social context in which student learning occurs. Health professions education (HPE) literature makes a strong case for understanding how students’ multiple social identities and the power structures within which they are trained influence learning (Rehman et al., 2023).

Research reporting students’ discomfort during IPE is common, related to issues of hierarchy that create feelings of marginalisation (Paradis & Whitehead, 2015). Encouraging students with different professional identities to interact with one another can create tension. For this reason disorientation is not unexpected; it is however unlikely that this tension is related to professional hierarchy alone (Hawick et al., 2021; Hutchings et al., 2013; Paradis & Whitehead, 2015). Few studies have explored the intersecting social identities of individuals learning with, from and about one another and how this influence their willingness to be actively engaged in IPE (Bochatay et al., 2022; Crampton & Afzali, 2021). It is time for research into IPE to shift attention away from the mere description of experiences to unpacking students’ lived realities. This includes exploring students’ intersecting social identities and biases that affect interaction among members of an interprofessional team (Crampton & Afzali, 2021; Tsouroufli et al., 2011).

Intersectionality provides a conceptual framework that aims to explore how multiple social identities can intersect and influence experiences such as privilege or oppression within existing social structures of power and exclusion (Crenshaw, 2018; Tsouroufli et al., 2011; Wilson et al., 2019). Although initially considered a framework to explore the marginalisation experienced by Black women, pioneered by Kimberlé Crenshaw (Crenshaw, 2018), intersectionality has now expanded to explore marginalisation and oppression across a wide range of social identities e.g. gender and ethnicity (Wilson et al., 2019). Intersectionality can offer a lens to explore students’ encounters with discrimination during IPE and help them understand the challenges interprofessional teams face related to individuals’ multiple intersecting identities in various contexts. Intersectionality is increasingly being used in HPE to understand and explore what other social identities or power differentials may be at play during students’ learning experiences (Crampton & Afzali, 2021; Tsouroufli et al., 2011). Understanding the influence of intersecting social identities, by students and educators, and how this affects their experiences of marginalisation, can facilitate a process to overcome the identified inequalities. This can take place through recognition, acceptance, reflection and action at an individual and systems level (Bochatay et al., 2022; Eckstrand et al., 2016; Rehman et al., 2023).

Acting on factors that result in inequitable learning experiences requires critical self-reflection and transforming the way individuals and institutions perpetuate systems of power (Rehman et al., 2023). Transformative learning is an integral learning theory adopted by institutions offering HPE globally (van Schalkwyk et al., 2019; Vipler et al., 2021). It is purported to foster students’ ability to reflect on, adapt and respond to the needs of society, act as agents of change, and broaden their understanding of the world (Mezirow, 1997). Transformative learning theory provides a framework that includes critical reflection for both students and educators, that can facilitate insight, meaning making and transformation when it comes to inequities and experiences of marginalisation.

Transformative learning theory describes an internal process of reflection, adaptation and integration after facing a disorienting dilemma. The theory is that this will result in the potential to effect change on a cognitive, affective and behavioural level with a resultant sustained transformation in action (Mezirow, 1997). The disorienting dilemma required to stimulate the process of transformation is likely facilitated when students are confronted with uncomfortable situations, but this must be followed by critical reflection. This process can take place in varying contexts that not only encourage critical reflection, but challenge students’ sense of worldview and understanding (Mezirow, 1997; van Schalkwyk et al., 2019). Transformative learning, as a theory, has a long history and the complexity and adaptations it has undergone over the years are not the focus of this paper (Lundgren & Poell, 2016; Kitchenham, 2008). We use transformative learning theory to explore how it can facilitate students’ (and educators’) processing of disorienting dilemmas of marginalisation, but also as a tool to guide educators in the development of transformative learning experiences in IPE. Understanding the theory of transformative learning as educators is crucial to recognise the processes that need to be facilitated to encourage students transformation journeys. Ensuring students move from disorientation, through reflection to reevaluating their and others’ role in the experience of learning is crucial in order for it to be impactful and ethical (Müller et al., 2024a). Insight into the process of transformative learning could result in an awareness of how educators can create more equitable IPE and subsequently improve patient care (Crampton & Afzali, 2021). To facilitate transformative learning, it is necessary to first understand what students are experiencing during undergraduate IPE and how intersectionality plays a role in their interprofessional learning experiences. Only then can educators understand, from their positions of privilege, how to role-model and adapt learning experiences to benefit interprofessional student engagement.

### Context

This study centres around two rural multiprofessional training platforms (RMTPs) in South Africa where students from four or more professions lived and worked together for anywhere from 8 weeks to 44 weeks of the academic year (Table 1). The majority of the students were housed on the campus or hospital grounds, but some students were placed 2–5 km away. Both RMTPs were more than 100 km from the central academic hospital and reliant on local clinicians and academic staff to facilitate clinical training. Both hospitals were regional hospitals in different provinces, with specialist services available to differing extents, with one being well-staffed and the other very understaffed. Each was the only referral hospital from district level services up to 400–600 km away. Each site had a weekly informal, non-credit-bearing IPE experience, described elsewhere (Muller, 2019).

As part of JM’s PhD study exploring the influence of rural longitudinal multiprofessional student placements on collaborative practice, formal and informal interprofessional learning opportunities were found to take place at curricular, co-curricular and extra-curricular levels (Müller et al., 2024b). The details of the students’ engagement and the duration of time they spent training on the platform have been explained separately (Müller et al., 2024b). During the analysis of the data for the aforementioned paper, individual factors such as language and religion were identified to play a role in the students’ abilities to engage interprofessionally, especially as it related to their experiences of marginalisation. These individual factors seemed to link to students’ social identities, for which we felt we needed a better understanding. Intersectionality as a theoretical framework provided us with insight into the complexity of IPE and interprofessional learning at the two RMTPs which are explored in this article.

This paper aims to share participants’ experiences of interprofessional learning on the RMTP through the lens of intersectionality and consider what strategies can be adopted to encourage equitable and transformative learning during IPE.

## Methodology

This exploratory qualitative research was part of a multiple case study research design using an interpretivist paradigm, conducted in 2022. This aspect of the study used a critical research paradigm to explore experiences of marginalisation.

### Data collection

Sixteen final-year undergraduate students from five professional degree programmes who trained for eight weeks or more on one of two RMTPs (previously described) in 2022 were purposively selected for this study (See Table 1). The five different health professions included Human Nutrition, Medicine, Occupational Therapy, Physiotherapy and Speech Language and Hearing Therapy. All students from the aforementioned programmes and sites were invited to participate via email (sent via their academic coordinators) and WhatsApp in October 2022. The student body at the time of participant selection consisted of approximately 70 students from both rural and urban origins from a variety of cities and towns across South Africa, a multitude of cultural backgrounds, language groups and ethnicities. Students were asked to self-identify their first language preference and cultural heritage to ensure a diversity of backgrounds and demographics to explore a wide range of experiences. Individual semi-structured interviews were conducted at the end of 2022 by JM using a semi-structured interview guide (Appendix A). Interviews were audio-recorded, anonymised and transcribed by JM prior to data analysis.

### Data analysis

An inductive thematic narrative analysis (Braun & Clarke, 2021) of the sixteen individual interviews was carried out to explore the influences on individuals’ lived experiences when it came to their interprofessional learning while living and working on one of the two RMTP. Data were read and re-read by JM and themes extracted from the data were charted and discussed with IC and EA. An independent researcher (AD) from a different professional and demographic background was invited to read the anonymised transcripts and inductively list apparent themes. AD and JM then engaged in a reflective discussion based on their individual themes and recognised that poetic inquiry may expose nuances in the data that they felt were lost in the extraction of quotes.

JM used a method of poetic inquiry called ‘Participant-voiced poetry’ in the next phase of data analysis, which involved creating poetic works directly from interview transcripts (Brown et al., 2021). Utilizing narrative inquiry opens a space for interpretation of perspectives and a deeper understanding of the data, but in some cases the presentation of data using quotation excerpts can be impersonal or dense, which van Rooyen and D’abdon (2020) argue may leave readers unmoved. When the goal of research is to contribute to both a cognitive and affective understanding of participants’ whole person experiences, literary tools such as poetry have been suggested as methods to enhance the goal of representation of meaning (Downie, 1991). Poetic inquiry works with, rather than against the complexities of experience, which researchers are always mining for understanding that is not easily extrapolated (van Rooyen & D’abdon, 2020). Poetry is also a literary tool that can be used to extend our insights into participant experiences and behaviour by encouraging continuous reflection on our understanding of data as researchers (Brown et al., 2021). Participant-voiced poetry, also known as ‘found poems’, does not add any words to the original transcript, but uses excerpts of an individual participant’s transcript that speak to a theme to create a poem (Brown et al., 2021; Glesne, 1997). JM extracted excerpts to construct the poems and discussed them with AD to explore any potential biases she may have had in representing the data. This process assisted with exploring underlying nuances she may have missed during the data analysis. JM then revised the poems based on AD’s feedback before sharing them with the individual participants whose data the poems were constructed from for their feedback and insights as a form of collective reflexivity or co-flexivity (Pithouse-Morgan et al., 2017). This practice enables co-composing and co-analysing poems as a form of reflection for researchers and participants and is discussed below.

### Positionality and reflexivity

All the authors are involved in HPE research and are qualitative researchers. Every author is from a different professional background, JM comes from a physiotherapy background, AD from social sciences, EA from nursing and ID medicine. Three of the authors (JM, EA and ID) are from similar cultural backgrounds, from the same ethnicity and all three studied in the health sciences. AD is from a different cultural heritage, ethnicity and profession affecting research team variance. Issues of reflexivity are discussed in the methodology. JM has been involved with practical IPE on the rural platform for 10 years and has insight into the types of learning activities taking place across the various professions at the sites where the students in this study were based. Her existing interaction and relationship with students as an interprofessional facilitator not involved in assessments, facilitated a deeper exploration of experiences and an iterative processes to data collection by recognising conflicting information and probing or rephrasing questions to reach a more comprehensive understanding (Cleland & Durning, 2015; Morse et al., 2002). Measures to ensure trustworthiness of the data collection and analysis are described below.

As much as individual’s intersecting social identities can influence their learning, so too can it influence a researcher’s interpretation of data. Each of us comes to the data through different backgrounds and it is important to note that there will always be bias because of the interaction between the data and the researcher (Wyatt et al., 2021). In an attempt to avoid reproducing inequity in this research we needed to be explicit about our potential biases (Abrams et al., 2020; Tsouroufli et al., 2011; Wyatt et al., 2021). In order to contribute to reflexivity and research team variance to help explore views from multiple perspectives, AD who is from a different demographic and professional background was purposively identified to engage in the anonymised data (Crampton & Afzali, 2021; Tsouroufli et al., 2011). To challenge her worldview and deepen data interpretation, JM also engaged in critical reflection with two independent researchers from different backgrounds related to her ethnicity and position of privilege, and how this may have influenced her interpretation of the findings.

The poems generated from the participant-voiced poetic inquiry were reviewed by AD who was given both the anonymised transcripts and voice recordings prior to reading the constructed poems. Acknowledging that the researcher is never passive but active in the role of interpretive work (Varpio et al., 2017) we wanted to member check our interpretation of the data. Participants were therefore asked to engage with the poems and comment on their relevance and interpretation. Participants were encouraged to re-write, critique, edit and translate the poems should they wish. The found poems were constructed with the explicit intent to remain faithful to the participants’ experiences, which is why co-creation and reflexivity with participants was so important. Entering into an open discourse and sharing, editing and co-creating poems with participants supports the trustworthiness of the data presented. The process of using found poems deepened our understanding of the complexities involved in making and sustaining relationships on the RMTPs (Pithouse-Morgan et al., 2017) and opened our eyes to the power of poetry in presenting affect in research and assisting participants as a form of reflective practice.

### Findings

The findings describe various individual experiences during interprofessional learning opportunities such as interprofessional peer-to-peer engagement, students’ engagement with qualified healthcare workers in the clinical environment and engagement with the patient or their family. Participants hailed from seven different cultural heritages (self-identified) and only three were first-language English speaking (self-reported), which is the official language used during training. Gender was not self-identified but ascribed based on the participants’ academic registration record with a 12:5 Female: Male ratio. The degree programme of the participants and the time spent training on the RMTP can be found in Table 1.

**Table 1:** Professional background and duration of placement of participants at RMTPs

Degree programmes and number of participants per programme	Approximate duration on RMTP during 2022
2 x HN^¶^	Jan - Nov (44 weeks)
4 x MBChB^‡^	Jan - Nov (44 weeks)
4 x OT^†^	13–40 weeks
4 x PT^*^	8 weeks
2 x SPT^§^	16 weeks in total at Site 1 and Site 2

^¶^ Human nutrition

^‡^Medicine

^†^Occupational therapy

^*^Physiotherapy

^§^ Speech, language and hearing therapy

The themes extracted from the data relate to the intersection of language and culture, representation, the intersection of being a student, at the bottom of the hierarchy and a profession that is considered less important that another, as well as the intersection of student and religion. In this paper, we present ten poems from eight participants which were selected from a rich plethora of experiences based on their relevance to this study, exploring intersectionality. Pseudonyms selected from common names associated with the self-identified cultural heritage of the participants were allocated by JM for each participant represented in the poems below.

### Connection and invisibility

The intersections of language and culture were raised by a number of participants as barriers to or facilitators of interprofessional engagement. This related to engaging with students, professionals or patients, which are all integral to the process of learning with, from and about each other in IPE. The poems are context-specific and relate to experiences of trust, connection, and (in)visibility stemming from language and culture. *Enzo* reflected that, despite being able to speak three of the twelve official languages in South Africa he was not able to speak to patients in their native language (Afrikaans). In a culture foreign to him *Enzo* reflected that this might affect his ability to care for his patients. He did however acknowledge his need to accept that this may be the norm for working in a culturally diverse country and would require adaptation on his part.

**Adapting to culture. (** ***Enzo*** **– Occupational Therapy)**

How am I going to connect to people?

You need that safe space

to connect

you want to hear them,

they need to trust you.

Now I can’t.

There’s no connection

To get the whole picture of who this person is

I am not Afrikaans

I’m stuck.

How can I keep patient beneficence?

To meet the population that’s here.

But

This is how the Community is,

what they speak is what they speak.

That realization

adapt

understand

I’m too self-centred - complaining

about Afrikaans

Zulu

Xhosa.

We are in South Africa

Move outside your environment

Come to that self-awareness,

Come to where you find yourself.

I was proud,

adapting to the culture here and how everyone is.

Another student, *Bafana*, had a limited comprehension of Afrikaans, one of the predominant local languages where he was working and was part of a student cohort training at a hospital in a predominantly Afrikaans speaking community. Consultants or clinical educators in this environment frequently declined to train students in English even though this was expected of them as educators. This practice subsequently affected *Bafana’s* ability to learn when other students and different professionals spoke in Afrikaans during ward rounds. *Bafana* reflects on the intersection he experienced in this hospital environment between feeling marginalised due to language which was ultimately tied to his racial identity. He explains how this affected not only his learning, but also his sense of being seen, which resulted in defensiveness and self-doubt.

**If you see me**,** then you will not need me to remind you I’m still here. (*Bafana*- Medicine)**

Ward rounds in Afrikaans,

expected me to go through it…

Speaking Afrikaans

because they don’t see us,

but

They see Them in that Afrikaans world.

They’re forming better bonds,

conducive to their learning.

  Racially biased?

Maybe it’s not about language anymore?

They don’t see Us, but they see Them.

For students of colour,

you feel you are being excluded.

  Racially biased?

immediately afraid to connect,

threatened

The human instinct is to be defensive.

Gaslighted.

It’s a thing if people don’t see you,

because if you see me,

you will not need me to remind you

  I’m still here.

### People like me

Experiences in the work place in terms of representation, especially in relation to ethnicity, were raised by participants who felt that this not only affected their interaction with patients but also their responsiveness to learning. *Thabo* shares his experience of how his ethnicity as an ‘African medical student’ (*Thabo-*Medicine*)* mattered in an encounter with a patient in a rural town and influenced his opportunity for learning and serving. This experience resulted in him leaving the situation, not knowing what to do and carrying the hurt throughout his final year. *Thabo’s* experience contrasts with *Irshaad’s*, who spoke only English. She, reflected on the value of clinician educator representation (people of colour) and language usage enabling a more conducive learning environment despite patients speaking a different language. The poems are placed in juxtaposition to one another below.

**Patient centred care (** ***Thabo*** **- Medicine)**

Old people.

They still live the old mentality,

One of them told me

up front

“Don’t touch me”

“You’re skelm” [sly]

“don’t touch me”

This young African doctor

“He’s black”

“I won’t speak to you”

She can speak English,

But won’t

“Speak Afrikaans,

I won’t say anything”

‘I’m going to try’.

I said,

Let’s speak.

Let’s try this.

I’m wasting my time.

She said.

‘Just go get the white doctor’

‘Go get the white nurse’

I left

I…

I just left.

**Representation (*Irshaad*– Speech Language and Hearing Therapy)**.

In XX hospital

patients were black,

they speak Afrikaans.

You work with it,

you understand them.

Doctors are people of colour.

Everything is done in English.

XX made me put my guard down.

Learning became easy.

### Voicelessness and belonging

Being a student working in a professional environment was perceived to influence participants’ opportunities to collaborate with other professionals and feel part of a team. Participants felt that ‘being a student’ came with little power of opinion working with qualified professionals, which in some cases was compounded by the existing professional hierarchy in the healthcare system. These experiences led to participants not feeling part of the team and affected their willingness to engage with other professions.

*Monika* reported that although she tried engaging with a qualified doctor on the ward round, there was not an opportunity to be heard and she attributed this to being a student studying human nutrition.

**Not the opportunity for confidence (** ***Monika*** **- Human Nutrition)**

During the ward rounds.

Speak up,

or not.

Voice your voice,

or not.

There’s not the opportunity

For confidence

You had to sit,

you had to see,

you had to listen.

Listen to the doctor.

You don’t feel like you’re part of the team.

Asking

“what’s your plan?”

There’s not really a response.

I feel

being a student…

It doesn’t mean much.

Voice your voice.

Never.

We are still training,

we’re not necessarily wrong.

It’s that hierarchy.

You don’t feel

part of the team.

There’s not the opportunity

For confidence

*Mari* reflected on her insecurity in engaging with medical personnel based on her perceptions of the professional hierarchy. She commented that as a student she did not understand how the system worked where she was training. She did however reflect on how her assumptions and insecurity potentially played a role in the perpetuation of hierarchy and the lack of interprofessional engagement.

**‘Me and my oversized scrubs’** (***Mari*- Physiotherapy)**.

I’ve never had a face-to-face conversation

with a doctor.

To discuss a patient.

Or explain a thing.

There’s no need,

you continue.

‘that’s your job’.

You sort your things out.

I’ll sort out mine.

There’s that knowledge gap

the friction is always there,

between what we do and what you do

doctors and allied health.

We don’t know the hierarchy,

don’t cross that line,

that magical line.

“We are doctors and you are Allied Health”.

Don’t want to ask questions

Don’t want to offend

Fear they think you don’t know better…

But I might have also contributed

to the thing.

Understand?

The typical situation,

me and my oversized scrubs

running around with crutches.

Oh, doctor, this patient…

A long-term problem

that mindset of

“Oh, we’re just the physios”

### The unification and exclusion of religion

The power of religious insight, sensitivity and access to religious communities in affecting interprofessional learning was evident. Religion intersected with participants’ identities as students who are part of the same ‘community’. The poems explore how their experiences affected their interprofessional engagement. Two poems by the same student who worked at both healthcare facilities that were the context of this study are presented below. These show her vastly different experiences engaging with students from different religions and professions at the two sites. The first poem reflects on a social occasion with students from different professions at the residence where she was staying at one RMTP. The second poem reflects on social interactions, at the other site, with students from other professions who already had pre-existing relationships with one another. In both accounts, *Irshaad* reflects on the differences in context regarding her ability to engage with students from other professions as a Muslim student.

**Two separate fires (*Irshaad*– Speech Language and Hearing Therapy)**.

Such a random group.

What was the university thinking?

But

It was nice to connect.

We saw each other as people.

I didn’t see you by your skin colour.

Religion didn’t play a part.

I didn’t see you by who you bow down to

or whether you bow down at all.

we have different beliefs.

doesn’t mean we can’t be in the same circle.

Everyone was so respectful.

‘Let’s have a braai’.

Two other Muslim students.

Two separate fires,

to not cross-contaminate our meat.

It didn’t limit us from engaging.

At the end [of our rotation] we all cried, saying goodbye.

Until today, we are still in touch.

**  Justifying who I am (*Irshaad*– Speech Language and Hearing Therapy)**.

Everyone comes from different walks,

different religions,

different ways of thinking.

But in XX,

It was a group of friends.

I was the only Muslim student.

I needed to always justify,

who I am.

I couldn’t do certain things.

“Please”.

“Don’t offer me a glass of wine”.

By.

the.

sixth.

week.

“Don’t offer me a glass of wine”.

I didn’t want to go out.

“You go ahead”.

I constantly explain myself.

I got tired of justifying who I am.

“You’re still offering me a glass of wine”.

*Phumeza* perceived the value of religion in bringing students from different professions together on the clinical training platform. The poem presents her acknowledgement of being from a different religion to her Muslim colleagues and her yearning to be part of the community they created.

**Building a community (*Phumeza*- Medicine)**.

Muslim students.

It’s beautiful,

it’s a beautiful thing.

More together

wherever they go.

Whatever they do,

always together.

Doesn’t matter what discipline.

they form this cluster.

they build a community.

Get to know each other.

Meet other disciplines.

Work together.

Share resources.

Maybe.

when you join them,

you never feel excluded.

Muslim students build a community.

I am a Christian

*Ana*, a Christian student attending a local church, was surprised by how her introduction to different professionals also based at the hospital through her attendance of the church enabled her to engage with them at work despite being a student.

**The church (*Ana*– Occupational Therapy)**.

Church

Students, clinicians

we did interact,

connecting

sharing

feeling comfortable

I could relate

social

professional

for the hospital

Get to know the person’s character

reach out

ask

listen.

I felt vulnerable,

I feel comfortable.

It’s isolating.

Not belonging,

Not belonging to some sort of religion

less opportunity to interact.

## Discussion

This paper provides a glimpse into the intersection of students’ social identities while training on a RMTP and how these experiences influenced student engagement in interprofessional learning opportunities. It is evident from the data that inequities in learning opportunities cannot be ascribed to biases related to one social identity alone e.g. profession. Intersecting identities such as ethnic representation, culture, language, religion, and their identity as a student also play a role in IPE and interprofessional learning. Experiences of marginalisation because of these intersecting identities are personal and emotional for students and deserve to be heard.

We, as health professions educators, need to consider why students are experiencing (and feeling) what they are and how these experiences are embodied by power structures in the training environment. Training contexts that appear neutral, may unfairly advantage some students and disadvantage others (Wilson et al., 2019). This can be the result of the inherent biases of student-peers, educators and patients, which may affect student learning and jeopardize equity in HPE (Crampton & Afzali, 2021). Students who ‘fit’ into a community in the training context may blend into the existing culture and practices at a training site and not be aware of the privilege and responsibility they have when it comes to facilitating inclusion and collaboration. Especially if they do not understand the influence other peoples’ intersecting identities may have in training contexts.

Power dynamics or structural hierarchies in training contexts where students train can also affect equity in HPE and need to be considered during training (Rehman et al., 2023). The healthcare environment is not always ideal, nor does it remain the same, and we will not always have control over it; however, understanding how the context influences student learning is important.

Take *Thabo’s* experience when the patient he was attending to, as a young African doctor (intersection of age, ethnicity and profession), refused his treatment based on the colour of his skin; it is evident that there is a complex interrelation between his social identities and the historical discriminatory practice of racism (Mokhachane et al., 2023). The intersection between individual and structural hierarchies is also evident in *Bafana’s* experience of language and ethnicity during ward rounds, which affected the quality of his training. Using intersectionality as a lens, the multidimensional axes of student identities become clearer, enabling institutions to explore structural contexts that may lead to students’ experiences of otherness that affect their engagement with peers, educators and patients. Marginalisation of health professional students during their training due to culture, ethnicity, language and other identities is not unique to South Africa (Mokhachane et al., 2023; Samuel & Konopasky, 2021; Simatele, 2022). A comprehensive exploration of student experiences and the overarching systems of power that influence them needs to be considered when choosing and developing training sites (Rehman et al., 2023). There needs to be an understanding of why power is perpetuated in certain systems and how this affects student learning, and an effort made by institutions to recognise and address structural inequalities in training environments (Crampton & Afzali, 2021; Eckstrand et al., 2016; Wyatt et al., 2021).

Intersectionality can offer the field of HPE an approach to identify and dismantle systems of social prejudice by recognising power, privilege and possibility through a process of reflection that can highlight identity and power differentials (Abrams et al., 2020). The first step in reducing discrimination requires individuals to be aware of their positionality regarding privilege and power. This requires a process of self-reflection and transformation to recognise their own biases, identify injustices and develop the desire to want to make a difference (Bochatay et al., 2022; Eckstrand et al., 2016). This may result in a certain level of discomfort or disorientation which can be the departure point for a transformative learning process.

We propose transformative learning as a theory in HPE to facilitate the development of students’ and educators’ responsiveness to the disorientations they may experience on the training platform. The transformative learning process can be a means to move beyond introspection, or what Mezirow would term non-reflective practice to a process of critical or premise reflection that results in a change in assumptions (Mezirow, 1991; Lundgren & Poell, 2016). We explore this below, but acknowledge that in order for transformative learning to be effective there needs to be a clear understanding by educator and students of the pedagogy and what it entails. This is crucial in facilitating the process of transformation beyond the disorienting dilemma and reflection to a place of profound transformation. In a previous paper we have detailed processes that can be introduced by faculties and educators to ensure an ethical transformative learning process so individuals are not stuck in what Kilgore and Bloom (2002) define as a halted transformation. Part of ethical transformative learning is ensuring that students (and educators) understand the pedagogy and process necessary for the transformation that hinges on a disorienting dilemma (Müller et al., 2024a).

Let us take *Enzo’s* disorienting experience of being placed in a training environment where he was unfamiliar with the culture and language of his patients, using Mezirow’s theory of transformative learning (Mezirow, 1991). In the process of his training, he recognised the dissonance between his competency and ability to connect to patients (content reflection). Following this he came to the realisation that he could not change his context (process reflection), but he can change how he thinks about and reacts to it if he wants to be a good therapist (premise reflection). *Enzo’s* processing of his disorienting experience, his reflection, re-examination and redefining of who he is in the world follows the interrelated process of transformative learning theory (Mezirow, 1997) which we can map alongside his poem (See Table 2).

**Table 2:** Mapping out a transformative learning experience using Mezirow’s theory of transformative learning (Mezirow, 1997)

Poem: Adapting to culture (*Enzo*)	Application of transformative learning theory process (Mezirow, 1997)
I am not Afrikaans	1) Disorienting dilemma
I’m stuck.	
How can I keep patient beneficence?	
To meet the population that’s here.	
But	3) Engage in assessment of assumptions
This is how the Community is,	
what they speak is what they speak.	
“…That realization	4) Recognise the process 5) Be open to exploring new roles
adapt	
understand	
…I’m too self-centred - complaining	2) Self-examination of feelings
Move outside your environment	5) Be open to exploring new roles 6) Plan a course of action 7) Knowledge and skills 8) Try on new roles
Come to that self-awareness,	
Come to where you find yourself.	
I was proud,	9) Build self-confidence
adapting to the culture here and how everyone is”.	8) Try on new roles 10) Integrate into the environment

The process of *Enzo’s* reflection moves beyond just focussing on the challenge of his disorientation and his intersecting identities (cultural differences and language barrier) but changes how he sees himself in the world, opting to take a new view on the situation. Mezirow’s additional eleventh step of “renegotiating relationships and negotiating new relationships” is not included in the above example as this did not form part of *Enzo’s* transformation trajectory at that point (Mezirow, 1991).

The transformative process that *Enzo* experienced is equally as important, if not more so for educators and people in positions of privilege, who witness the disorientation of or perpetrate prejudice and marginalisation during training. Let us reflect back to *Bafana’s* poem, *‘If you see me*,* then you will not need me to remind you I’m still here’*. If colleagues who witnessed his distress during the Afrikaans ward rounds critically reflected on his experience and moved beyond their discomfort to a place of seeing injustice and wanting to advocate for change, would *Bafana’s* experience have been different? Understanding the value and process of the transformative learning pedagogy may provide a useful tool to help students (or educators) shift into action. Rehman et al. (2023) promote the development of this critical consciousness of intersecting identities to become aware of inequalities in social relationships so that something can be done about it. Critical reflection and conscientizing should however take place at the individual level, in the training context and in the health care environment. Reflective practice can include students, educators and faculty management in analysing the interplay between their own social identities and those of others as well as how power and inequity are fostered in certain environments.

Students and educators who critically reflect on existing power differentials and biases affecting learning experiences because of an intersectional analysis could introduce and adopt mechanisms to curb marginalisation. Going back to *Bafana’s* experience on the ward round, had his peers, educators or other professionals reflected on their privilege of understanding the adopted language in that context and acknowledged the exclusion of *Bafana*, his learning experience would likely have been very different. Students or educators who reflect critically may recognise potentially isolating environments or notice discriminatory experiences their peers might have and avoid falling into the trap of ‘sticking with my people’ because it is comfortable; Eckstrand et al., (2016) refers to this process as embracing personal and collective loci of responsibility.

It is clear in *Irshaad’s* experiences on the two platforms that interprofessional peers on the one platform had the insight to make provision for her to be involved in a social meal while other students repeatedly disregarded her religious practices. Interventions such as workshops on cultural competence should be accompanied by community-oriented training programmes (Eckstrand et al., 2016), skills development for critical reflection (Bochatay et al., 2022; Rehman et al., 2023) and an understanding that identities intersect and need to be acknowledged and considered when engaging in learning. It is also clear from the findings in this article that professional hierarchy can no longer be the singular focus of students’ IPE experiences and the multidimensional intersecting identities of students need to be considered in the interprofessional context.

We reflect, as authors, on the disorientation we experienced exploring participants’ stories and poems and recognise that our process of self-reflection and insight can be best understood as a process of transformative learning. The process of poem creation, reflection and discussion with participants was deeply moving and found poems has opened our eyes to a valuable methodology to give participants’ a voice. The feedback was overwhelmingly positive during email and WhatsApp correspondence with the participants regarding the poems constructed from their data. One participant commented *‘The poem spoke to me much. I could not have described my journey better’*. (email from *Bafana*) Another participant reflected *‘I don’t think you could have put it in a better way. I actually let one of my colleagues read it as well… she actually started crying… definitely*,* I do feel that with compiling the poems*,* it kind of did bring out the emotional side*,* which I think that is what you were aiming to do. Yeah*,* I wouldn’t have changed anything’* (conversation with *Irshaad*).

We can see that exploring views and experiences from multiple dimensions by ensuring research team divergence is important to not be bound by our assumptions (Crampton & Afzali, 2021; Wyatt et al., 2021). Ultimately as educators, researchers and students alike we need to reflect on who we are and how we became this way, and we need to analyse how our identities and contexts affect our experiences. We also need to be open to hearing about and being affected by the experiences of others, and acknowledge our connection to systems of privilege and disadvantage.

### Limitations

We recognise that similar experiences of marginalisation could be had in urban environments and are not specific to only one context; thus we believe the insights from our research could help educators mitigate for biases and out-group experiences students may have in any training environment.

We are cognisant that the majority of the research team has limited personal experience of marginalisation related to ethnicity, language or culture based on our historical and current privilege and we acknowledge that this would affect our interpretation of the data. The methods of reflexivity included in the process of this study have broadened our perspectives and given us new insights into privileges and power. We believe the process of critical conscientizing with the facilitation of our co-researcher, critical friends and research participants required us to look at the data and the intersection of our own social identities very differently. We acknowledge the limitations of the theory of intersectionality and understand that multiple alternative theories exist that could be used to explore the data such as critical race theory, feminist theory or post-colonial theory.

Abrams et al. (2020) caution against using intersectionality only after study conceptualization as a theory to analyse data, much like we did, arguing rather that it should guide the research study design, generate important considerations for interactions with participants during recruitment, data collection, and dissemination (Abrams et al., 2020).

Mezirow’s theory of transformative learning is complex and has been adapted and revised over the years, including by Mezirow himself (Kitchenham, 2008; Lundgren & Poell, 2016). He suggested four types of learning and three types of reflection as well as added to his 10-step framework; these have not been explored in this paper, but could be a useful framework to analyse the process of reflective practice and transformation that the students are undergoing. Exploring students’ content, process and premise reflection in facilitating perspective transformation is important if we want students (or educators) to get to a point of ‘profound transformation’ (Mezirow, 1991). By assessing the level to which participants reflected in this study and exploring indicators of reflection, we can enhance the potential for critical learning opportunities in IPE (Lundgren & Poell, 2016). Using found poems or self-authored poems as a reflective process during training may be a useful tool to explore and facilitate premise reflection and perspective transformation and further investigation is recommended. Research using found poems or poetic construction of reflection may facilitate the investigation of emotional aspects and affects in conjunction with cognitive learning, which has been called for in research on reflection (Lundgren & Poell, 2016).

## Conclusion

In this paper, we shift attention away from merely describing experiences of IPE and unpack the interconnections and complexity of differences when students, professionals and patients come together with the potential of learning with, from and about each other. By using intersectionality as a framework, we have gained a nuanced understanding of how various social identities relate to one another in the context of learning on the RMTP. Knowing that collaborative engagement is so heavily dependent on relationships, educators and students must be cognizant of the influences of their social identities and the discomfort they and others may experience in certain contexts. We propose using transformative learning theory to help students and educators become more aware of the complexity of intersecting social identities, context and power differentials in learning and in IPE. By understanding the intersecting factors that influence IPE in HPE, educators may be able to create more equitable opportunities for students to collaborate and learn from their interprofessional peers.

## Electronic supplementary material

Below is the link to the electronic supplementary material.

Supplementary Material 1

## Data Availability

The qualitative data that support the findings of this study are not publicly available to maintain confidentiality. Anonymized data however, may be available from the authors upon reasonable request and if agreed to by the authors affiliated institution.
