# Supplementary material for: Exploring poems of intersectionality in the disorientation of interprofessional learning
Source: Adv Health Sci Educ Theory Pract. 2025 Mar 31;30(5):1599–620. doi: 10.1007/s10459-025-10428-5 (PMC12572009; doi:10.1007/s10459-025-10428-5)
Supplement: Supplementary file 1 — Supplementary Material 1 [file 10459_2025_10428_MOESM1_ESM.docx]

**Appendix A: Semi-structured interview guide**

1. Where have you been working this year? How has it been?
2. What is your understanding of collaborative patient care?
3. What is your experience of collaborative patient care where you have been working?
4. Describe some of the experiences you had of working or engaging with professionals from other disciplines this year?
   1. What were some of the things that made these engagements possible?
   2. What limited these engagements / interactions with other professionals?
   3. Does your professional role as e.g., Doctor/ Physiotherapist play any role in this?
5. How would you perceive your ability to collaborate professionally to improve patient care and health systems during this year?
   1. Can you give me an example?
   2. What made his possible?
   3. What limited this?
6. You mentioned ……………………. last year during our interview as a ……………. to being able to work collaboratively with students from other professions. What do you think about those experiences now in hindsight? And do you still feel that they ……………… you in the same way?
7. What aspects of your undergraduate training stand out as having been of greatest benefit in terms of your preparedness to manage patients collaboratively?
8. What aspects of working interprofessionally to improve patient health did you feel least prepared for during this year?
9. Looking back, what do you think could have been different during your undergraduate training to better equip you to work interprofessionally to improve collaborate patient care as a healthcare professional in South Africa?
